# The ACTIVE study protocol: apatinib or placebo plus gefitinib as first-line treatment for patients with EGFR-mutant advanced non-small cell lung cancer (CTONG1706)

**DOI:** 10.1186/s40880-019-0414-4

**Published:** 2019-11-07

**Authors:** Zhonghan Zhang, Fan Luo, Yang Zhang, Yuxiang Ma, Shaodong Hong, Yunpeng Yang, Wenfeng Fang, Yan Huang, Li Zhang, Hongyun Zhao

**Affiliations:** 1 State Key Laboratory of Oncology in South China, Collaborative Innovation Center for Cancer Medicine, Guangzhou, 510060 Guangdong P. R. China; 2 Department of Medical Oncology, Sun Yat-sen University Cancer Center, 651 Dongfeng Road East, Guangzhou, 510060 Guangdong P. R. China; 3 Department of Clinical Research, Sun Yat-sen University Cancer Center, 651 Dongfeng Road East, Guangzhou, 510060 Guangdong P. R. China

**Keywords:** NSCLC, EGFR, VEGFR, Tyrosine kinase inhibitors, Apatinib, Gefitinib, Randomized, Double-blind, Placebo, Phase III

## Abstract

**Background:**

Gefitinib, as the first epidermal growth factor receptor tyrosine kinase inhibitors (EGFR-TKI) approved for the treatment of advanced non-small cell lung cancer (NSCLC), has been proved to significantly improve the progression-free survival (PFS) in the first-line setting but suffers from resistance 7–10 months after treatment initiation. Apatinib (YN968D1), a potent vascular endothelial growth factor receptor (VEGFR) 2-TKI, specifically binds to VEGFR2 and leads to anti-angiogenetic and anti-neoplastic effect. Concurrent inhibition of VEGFR and EGFR pathways represents a rational approach to improve treatment responses and delay the onset of treatment resistance in EGFR-mutant NSCLC. This ACTIVE study aims to assess the combination of apatinib and gefitinib as a new treatment approach for EGFR-mutant NSCLC as a first-line setting.

**Methods:**

This multicenter, randomized, double-blind, placebo-controlled phase III study (NCT02824458) has been designed to assess the efficacy and safety of apatinib or placebo combined with gefitinib as a first-line treatment for patients with EGFR-mutant advanced NSCLC. A total of 310 patients with EGFR-mutation (19del or 21L858R), pathological stage IIIB to IV non-squamous NSCLC were to be enrolled. The primary endpoint is investigator assessment of PFS, and the secondary endpoints include independent radiological central (IRC)-confirmed PFS, overall survival (OS), objective response rate (ORR), disease control rate (DCR), time to progressive disease (TTPD), duration of response (DoR), quality of life (QoL) and safety. The patients are randomized in a 1:1 ratio to receive gefitinib (250 mg, p.o. q.d.) plus apatinib (500 mg, p.o. q.d.) or gefitinib plus placebo, given until disease progression or intolerable adverse events. Exploratory biomarker analysis will be performed. This study is being conducted across China and comprises of 30 participating centers. Enrollment commenced in August 2017 and finished in December 2018, most of the patients are in the follow-up period.

**Anticipated outcomes and significance:**

The present study will be the first to evaluate the efficacy and safety profile of the combination of apatinib plus gefitinib as a first-line therapy for patients with EGFR-positive advanced non-squamous NSCLC. Importantly, this trial will provide comprehensive evidence on the treatment of EGFR-TKIs combined with antiangiogenic therapy.

*Trial registration* Clinicaltrials.gov NCT02824458. Registered 23 June 2016

## Background

Lung cancer remains the leading cause of cancer-related mortality worldwide, with a 5-year survival rate of less than 20% [1–3]. Mutations in the tyrosine kinase domain of epidermal growth factor receptor (EGFR) had been identified as a significant subtype of non-small cell lung cancer (NSCLC) [2, 4], present in about 25%–50% of advanced NSCLC patients in Asia [1]. The emergence of EGFR-tyrosine kinase inhibitors (TKIs) for the treatment of lung cancer has significantly changed the therapeutic landscape of NSCLC in the past few decades. Gefitinib, as the first EGFR-TKI approved for the treatment of advanced NSCLC after failure of chemotherapy, has been proved to significantly improve the progression-free survival (PFS) of first-line setting compared with standard chemotherapy in NSCLC patients harboring the EGFR mutation, by series of large-scale phase III clinical trials, i.e. the IPASS [5], WJTOG3405 [6], and NEJ002 trials [7]. However, a majority of patients relapse or experience progressive disease (PD), 9–13 months after treatment initiation with the first-generation EGFR-TKIs [5–8]. To improve survival and response duration, exploring strategies for delaying EGFR-TKIs resistance has become an urgent challenge.

Recognition of tumor vasculature normalization is becoming a new antiangiogenic therapeutic concept for cancer treatment [9, 10], while the vascular endothelial growth factor receptor (VEGFR) signaling pathway (especially the VEGFR-2) act importantly in controlling the regulation to tumor vascular function [11, 12]. A previous study also showed that an overactive VEGFR pathway independent of EGFR may play a role in the observed resistance to EGFR-targeted therapies [13]. Functionally, dual blockade of both tumor (EGFR signaling) and the endothelial cells (VEGFR signaling) that support tumor growth may ultimately make a difference in the effectiveness of cancer treatment [14, 15]. Also, the toxicity profile of anti-EGFR therapies differs from that of anti-VEGFR therapies [15], making the combination strategy possible to be tolerated by lung cancer patients. The dual blockade of both EGFR and VEGFR signaling seems to be a promising treatment strategy with a synergistic combination effect [16, 17].

Dual inhibition of both VEGFR and EGFR has shown efficacies in delaying the emergence of resistant tumors in many preclinical and early clinical studies [17–19], which provides a strong rationale of combined treatment with anti-VEGFR and anti-EGFR therapies. Several clinical trials also support the idea of combined inhibition of both the VEGFR and EGFR pathways to promote more beneficial anti-tumor effects. A single-arm, phase II trial, the BELIEF study, demonstrated a 1-year PFS of 55.6%, with a median PFS of 13.6 months of erlotinib plus bevacizumab in EGFR mutated NSCLC patients [20]. The JO25567 study evaluated bevacizumab plus erlotinib in advanced non-squamous NSCLC patients with activating EGFR mutations [21], showing that a PFS improvement of 6.3 months was achieved in the erlotinib plus bevacizumab group when compared to erlotinib monotherapy group (16.0 months vs. 9.7 months, hazard ratio [HR], 0.54, 95% confidence interval [CI] 0.36–0.79, *P *= 0.0015). The treatment was well-tolerated and serious adverse events were similar between the two groups. Similar results were reported from a phase III NEJ026 study [22]. Except for these studies, several ongoing clinical trials such as the RC1126 ACCRU (NCT01532089) and the BEVERLY (NCT02633189) trial are also investigating the efficacy of erlotinib plus bevacizumab regimen.

Small-molecule multi-targeted TKIs have become the focus of attention in the field of targeted therapy in recent years. As a potent VEGFR2-TKI, Apatinib (YN968D1) is a novel targeted agent developed and manufactured in China, also the world’s first small molecule antiangiogenic targeted agent verified to be efficacious and safe for gastric cancer. Apatinib was found to significantly improve the overall survival (6.5 months vs. 4.7 months; *P* = 0.0149; HR, 0.709; 95% CI 0.537–0.937; *P* = 0.0156) of patients with advanced gastric cancer after failure of frontline standard chemotherapy, which was approved by the China Food and Drug Administration (CFDA) in 2014 [23, 24]. Several in vivo and in vitro studies have shown that apatinib can inhibit the proliferation of lung cancer cells [25–27]. A multicenter randomized placebo-controlled phase II study on 135 advanced NSCLC patients after two lines of treatment failure found that apatinib had substantial clinical activity without significant additional toxicity [28]. Promisingly, in our exploratory phase I study evaluating apatinib plus gefitinib as a first-line of treatment for NSCLC, of the twelve evaluable patients, the observed objective response rate (ORR) was 83.3% (10/12), and disease control rate (DCR) was 91.7% (11/12). The observed median PFS was 19.0 months in the apatinib (500 mg) plus gefitinib group while a PFS of 13.4 months was achieved in the apatinib (250 mg) plus gefitinib group (*P* = 0.657) [29]. Simultaneously targeting the EGFR and VEGR pathways might be a feasible therapeutic approach for patients with EGFR-mutant NSCLC. Therefore, here we describe this phase III ACTIVE study to assess the efficacy and safety profile of apatinib combined with gefitinib as first-line treatment in advanced NSCLC patients harboring activating EGFR mutations.

## Methods

### Study design and objectives

**Fig. 1:**
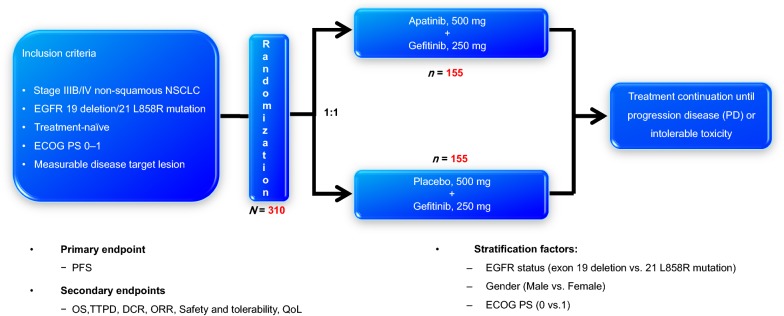
Graphical representation of the study design of this ACTIVE (CTONG1706) study. *NSCLC* non-small cell lung cancer, *EGFR* epidermal growth factor receptor, *ECOG PS* Eastern Cooperative Oncology Group performance status, *OS* overall survival, *ORR* objective response rate, *DCR* disease control rate, *TTPD* time to progressive disease, *QoL* quality of life, *PD* progression disease

This is a multicenter, randomized, double-blind, placebo-controlled phase III study of apatinib or placebo plus gefitinib as a first-line of treatment for patients with EGFR-mutant advanced NSCLC. Preliminary stratification factors including EGFR mutation status (exon 19 deletion, 21 L858R mutations), gender (male, female) and PS score (0, 1) (Fig. 1). The double-blindness procedure is maintained during the process of the clinical trial. Identical appearance of simulation agent was produced and personnel in the participating centers, participants, or investigators involved in this clinical trial are masked to the intervention allocation of each participant according to double-blind procedures. The study is being conducted in 30 centers across China. Clinical trial information: (ACTIVE study: Apatinib Combined with EGFR-TKI In Treatment-naive EGFR-mutant NSCLC patients).

### Randomization and blinding

Patients were centrally randomized, using BioKnow-RTSM managed by Beijing BioKnow Information Technology Co. Ltd., Beijing China, and allocated in 1:1 ratio to an experiment arm or a control arm with the use of minimization method as described by Pocock and Simon [30, 31]. The balance of stratification factors (importance ranking in descending order) including EGFR mutation sites (exon 19, 21), gender (male, female) and PS score (0, 1) is ensured. The patient is required to receive the allocated drug treatment within 48 h after randomization.

The placebo agent has an identical appearance to the experimental drug. Random assignment forms were generated by using the Statistical Analysis System (SAS) software, (SAS Institute, Cary, North Carolina, USA) PROC PLAN from Beijing BioKnow Information Technology Co. LTD. Study drugs are coded and loaded into the central randomization system with random allocation schedule as blind codes. Personnel in the participating center, patients, sponsors, and staff involved in the clinical trial are masked to the treatment regimen of each patient.

### Treatment administration (including both apatinib/placebo and gefitinib)

Apatinib (500 mg orally, once daily; Jiangsu Hengrui Medicine Co. Ltd., Lianyungang, Jiangsu, China) or matching placebo (Jiangsu Hengrui Medicine Co. Ltd., Lianyungang, Jiangsu, China,), in accordance with the randomization schedule, were administered. A cycle of treatment is defined as 28 days of one daily dose of apatinib/placebo.

Group A (experimental arm): gefitinib 250 mg (Qilu Pharmaceutical (Hainan) Co., Ltd., Haikou, Hainan, China), p.o. q.d. + apatinib 500 mg, p.o. q.d.; on an empty stomach (preferably at the same time every day), every 4 weeks.

Group B (control arm): gefitinib 250 mg, p.o. q.d. + apatinib simulation agent (placebo), 2 tablets, p.o. q.d.; on an empty stomach (preferably at the same time every day), every 4 weeks.

The initial dose for apatinib is 500 mg, p.o. q.d., every 28 days. Combination therapy is given until disease progression, intolerable adverse events, patient withdrawal of informed consent or treatment discontinuation at the discretion of the investigator. Patients who discontinue apatinib due to intolerable adverse events are allowed to switch to gefitinib until disease progression. In principle, patients with PD need to stop the study treatment. However, considering the treatment characteristics of molecule targeted agents, for patients with remarkable necrosis or degeneration within the intralesional structure, consistently improved or stable clinical symptoms related to tumor, the treatment is allowed to continue under close observation until unacceptable adverse events or recurrence of PD, provided the patients voluntarily willingness to continue the medication and that there is anticipated beneficial survival effects for the treatment continuation; at the discretion of the principal investigator.

Predefined dose modification and discontinuation were permitted to manage treatment-related adverse events. When patients experienced grade 3/4 hematological toxicities, dose suspension is considered for grade 3 toxicities, and dose reduction is done for grade 4 toxicities until the toxicity became tolerable, at grade 2 or less. For grade 2 non-hematological toxicities, doses were suspended until the toxicity became tolerable according to the judgment of investigators. Additionally, when grade 3/4 non-hematological toxicities occurred, dose suspension is performed, and dose re-administration is given, at a lower level of the initial dose, when the toxicities became grade 1 or less. However, when patients experienced grade 3/4 toxicities again, they immediately discontinued the study treatment to receive a predefined treatment modality. Additionally, patients requiring treatment interruption for more than 14 days (either continuously or cumulatively) or more than two times in a defined treatment cycle were withdrawn from the study treatment.

### Key eligibility criteria

Patients between the age of 18 to 75 years old who are diagnosed with stage IIIB/IV non-squamous NSCLC harboring activating EGFR mutation (exon 19 deletion or L858R point mutation in exon 21), with an Eastern Cooperative Oncology Group (ECOG) performance status (PS) ≤ 1, adequate organ function, and no history of prior chemotherapy or other targeted therapy, were eligible for inclusion in the trial (full patient inclusion and exclusion criteria are detailed in Table 1).

**Table 1:** Patient inclusion and exclusion criteria

Key inclusion criteria
1. Age of 18–75 years at enrollment
2. Eastern Cooperative Oncology Group (ECOG) performance status (PS) of 0–1
3. Life expectancy of ≥ 12 weeks
4. Histologically confirmed stage IIIB (unsuitable for radiotherapy) or IV non-squamous NSCLC, with measurable tumor lesions (on computed tomography [CT] scan according to the Response Evaluation Criteria in Solid Tumors [RECIST] 1.1)
5. Primary NSCLC harboring activating EGFR mutation (exon 19 deletion or L858R point mutation in exon 21) confirmed with any validated methods, and the mutation detection required to be undergone prior to the trial in newly diagnosed IIIB-IV non-squamous NSCLC
6. No history of prior chemotherapy or other targeted therapy
7. Prior radiotherapy allowed to < 25% of the bone marrow (Cristy and Eckerman 1987)
8. Adequate organ function (hematological, renal, hepatic, coagulation)
9. Participants joining the trial based on the personal decision, providing written informed consent, with good compliance to treatment and follow-up
Key exclusion criteria
1. Symptomatic brain metastases
2. Imaging assessments (with CT or MRI) showing the distance of tumor to major blood vessels ≤ 5 mm, or presence of central tumor invasion of local major blood vessels; or evidence of pulmonary cavitary or necrotic tumor
3. Patients with hypertension under ongoing treatment with 2 or more antihypertensive agents
4. Detection of ALK fusions or T790M mutation positive by gene testing
5. Presence of cardiovascular diseases; coexistence or history of interstitial lung disease; abnormalities of coagulation test; presence of clinically problematic bleeding disorders or significant bleeding tendencies; presence of documented arterial and venous thrombotic events and so on
6. Hemoptysis with 2 teaspoons or more of bloody sputum every day before enrollment
7. Urine test showing urine protein ≥ ++, or evidence of 24-h urine for total protein ≥ 1.0 g
8. Pregnancy or lactation; reproductive-age women unwilling or unable to use an effective contraceptive method

### Follow-up

Imaging assessment for efficacy is conducted after the 1st cycle (28 days) and the following assessment is performed on every 2-cycle (56 days). Imaging assessment (including chest, abdomen, and brain CT or MRI) during treatment period are required to be performed under the same condition similar to baseline, to review the chest, abdomen or other lesions identified at baseline; suspected new lesions require timely evaluation. Imaging studies are required to be performed at the time of participant withdrawal for any reason. Imaging studies schedule allows an extended window (± 7 days). Unscheduled imaging studies are suggested in cases where disease progression is suspected (for example, symptomatic deterioration). For participants who stop study treatment for reasons other than disease progression confirmed by imaging modalities, imaging assessment is required at the time of study discontinuation (if there are no imaging studies within 4 weeks before study discontinuation) Follow-up for survival outcome is performed in cases of study treatment interruption, survival, and subsequent antineoplastic therapy data is collected by clinical or telephone follow-up every 3 months until the patients death.

### Study end points and assessments

The primary outcome is PFS assessed by the investigator, which is defined as the time from randomization to the first time of disease progression or death from any cause, whichever comes first. The secondary outcomes include a sensitivity analysis of PFS performed by blinded independent central review, OS, which is defined as the time from random assignment to death from any cause, ORR, DCR, time to progressive disease (TTPD), duration of response (DoR), quality of life (QoL). Objective treatment response is evaluated by computed tomography according to the Response Evaluation Criteria in Solid Tumors (RECIST) version 1.1 and classified as complete response (CR), partial response (PR), stable disease (SD) and progressive disease (PD). PFS is also assessed by an independent radiological central (IRC) review of radiological data from all randomized patients with at least a baseline and on the 8-week follow-up scan by masked independent radiologists. The safety of the combined therapy is evaluated by recording the incidence of severe adverse events (SAEs) and adverse events (AEs) according to the National Cancer Institute Common Toxicity Criteria (NCI-CTC) version 4.0. In this study, any adverse medical event occurring from initiation of the experimental drug up to 30 days after the last dose of the experimental drug, whether or not considered related to the experimental drug, is defined as AE. SAEs are defined as death, illness requiring hospitalization, events deemed life-threating, events that result in persistent or significant disability or incapacity, a congenital anomaly or birth defect, or other important medical conditions. QoL refers to EORTC QLQ-C30 (version 3, Chinese version) and EQ-5D (Chinese version). Patients are scored based on changes of related clinical symptoms and examinations observed before and after the treatment, and scores produced in different domains are entered into electronic case report form (eCRF) according to QoL scale instruction.

### Data collection and management

eCRF is deployed to collect data in this study. Each individual involved in conducting data collection is qualified by training to perform tasks on the electronic data capture (EDC) system (Beijing Bioknow Information Technology Co., Ltd.).

The principal investigator or designated data entry clerk (CRC) should enter the patients’ data into the EDC system in accordance with the follow-up requirement and eCRF completion guide. Logical verification procedure implemented in the system typically runs data check to assure the completeness and logicality of the study related records in EDC system and return with, if any, the tooltip of eCRF entry error, and principal investigator or the CRC can make appropriate corrections and explanations (if necessary). The principal investigator will get CD-ROM or certified copy containing the patient data after the database is locked for record-keeping. eCRF is required to be signed by the investigator or the investigator’s designated representative and data recorded in eCRF needs to be verified to ensure credibility. Any change or correction to an eCRF and source documents should be dated, signed, and explained (if necessary), and should not obscure the original entry.

### Sample size and statistical considerations

The primary endpoint is the investigator assessment of PFS in this study. By consulting relevant clinical trials and literature [6–8, 26, 27], median PFS in the control arm (placebo + gefitinib) is about 10 months, and expected median PFS in the experimental arm (apatinib + gefitinib) is prespecified as 15 months (difference in PFS up to 5 months is considered as clinically significant). Superiority test is performed in this study with the purpose of addressing whether the experimental treatment is better than the control. Using a significance level α = 0.05 (two-tailed), 1 − β = 0.80, median PFS in experimental arm and control arm are 15 and 10 months, and the ratio of sample size in two arms is 1:1. Recruitment is to be completed in 18 months, follow-up carried on in 18 months. Using the PASS (Power Analysis and Sample Size) 2015 Statistical Software (UT, USA). that based on Group-Sequential Log-rank Tests (Simulation) to calculate the sample size, at least 124 of patients in each arm is needed. The total number of patients to have PFS events in the two arms is expected to be 192 (88 in the experimental arm, 104 in control arm). Taking a 20% drop-out rate into account, the sample size for experimental arm and control arm should be at least 155 each for a total of 310 enrolled patients.

The trial is being conducted throughout China with 30 participating hospitals as sub-centers, in accordance with the Declaration of Helsinki, the International Conference on Harmonization Tripartite Guideline for Good Clinical Practice, and applicable region-specific requirements. The trial will be initiated only after approval by the respective institutional review boards/independent ethics committees at each center. All patients must provide written informed consent.

### Statistical analysis

All statistical analyses are to be carried out using the SAS software (version 9.3 or above, SAS Institute, Cary, North Carolina, USA). All statistical tests are two-tailed, and *P* ≤ 0.05 is considered to indicate statistical significance, with a 95% confidence deployed interval. For quantitative data, such as age, height and weight, their respective mean, standard deviation, median, maximum, and minimum are calculated. For qualitative data, such as gender and ECOG score, their frequency and percentage are calculated. For the primary endpoint, the Kaplan–Meier method is applied for the PFS curve with estimation for median and 95% CI. Survival differences are compared between the groups with a log-rank test. Cox regression model, stratified according to factors such as EGFR mutation sites (19 or 21), gender and PS, is used to perform between-group survival comparisons, calculate the adjusted hazard ratios (HR) and 95% CI. For secondary endpoints including OS, TTPD, and DoR, event-time distributions are generated by Kaplan–Meier method with estimation for median and 95% CI. Survival differences are compared between groups with the log-rank test; disease control rate and overall response rate and 95% CI are calculated, and between-group survival comparisons are conducted using CMH or χ^2^ test; independent sample t-test or Wilcoxon rank-sum test is employed to compare between-group QoL score. For safety analysis, descriptive statistical analysis is applied, and mean or rate of laboratory examination results before and after treatment are compared when required. Vital signs, workup, and laboratory evaluation are used to describe changes before and after treatment, and potential correlation between abnormal findings and experimental drug; Medical Dictionary for Regulatory Activities (MedDRA) is used to standardize all adverse events according to system organ class (SOC), preferred terms (PT) and Common Terminology Criteria for Adverse Events (CTCAE), and to compare incidence of adverse events and grade 3 or above adverse events.

## Discussion

The advent and use of EGFR TKIs have revolutionized treatment for advanced lung cancer. The EGFR TKIs such as gefitinib and erlotinib have been approved for the treatment of activating EGFR mutation-positive NSCLC. However, most NSCLC patients with activating EGFR mutation will develop resistance and relapse after 9–13 months’ initial treatment of EGFR-TKIs [5–8]. Thus, to overcome the current resistance of EGFR-TKIs is of paramount importance. It has been indicated that the EGFR pathway is synergized and influenced by the VEGFR pathway signaling. A preclinical study has shown that EGFR activation can upregulate the VEGFR signaling pathway by increasing the production of VEGF in human cancer cells [18]. In contrast, EGFR blockade suppresses the secretion of VEGF and other angiogenic growth factors [32] and inhibition of EGFR or its downstream effector mTOR can also downregulate the VEGF and inhibit capillary formation by endothelial cells [33]. Dual inhibition of both VEGFR and EGFR has been shown in delaying the emergence of resistant tumors in many preclinical and early clinical studies, which provides a strong rationale for the combination strategy of anti-VEGFR and anti-EGFR therapies [15, 17, 19].

A single-arm phase II trial, the BELIEF study, demonstrated a 1-year PFS of 55.6%, with a median PFS of 13.6 months of erlotinib plus bevacizumab in EGFR mutated NSCLC patients [20]. An open-label randomized multicenter phase II study (JO25567) evaluated bevacizumab plus erlotinib in patients with stage IIIB/IV or recurrent non-squamous NSCLC with activating EGFR mutations [21]. The median PFS was 16.0 months in the bevacizumab plus erlotinib group vs 9.7 months in the erlotinib monotherapy group (HR, 0.54, 95% CI 0.36–0.79, *P *= 0.0015). The BELIEF [20], JO25567 [21] and NEJ026 [22] study have shown great improvement of PFS with this dual inhibition of VEGFR and EGFR strategy [21, 22], indicating the significant potential of reversing resistance developed by EGFR-TKIs monotherapy. There are several ongoing clinical trials examining the efficacy of erlotinib and bevacizumab, such as RC1126 ACCRU (NCT01532089) and BEVERLY (NCT02633189). However, in the regimen mentioned above, bevacizumab is administered intravenously every 3 weeks in the hospital, which is not so convenient for the patients and might cause hypersensitivity. Therefore, better regimens are to be explored to help promote the convenience and comfort of patients. Apatinib is an oral VEGFR-TKI that has a potent effect on lung cancer. Monotherapy using apatinib has shown substantial anti-tumor activity and tolerable toxicity profile in a phase II study [28]. A recent preclinical study showed that in H1975 tumors transplanted mice model, the expression of Ki-67 and proliferating cell nuclear antigen (PCNA) were significantly decreased after the intervention of apatinib plus gefitinib compared with gefitinib or apatinib alone, indicating that the addition of apatinib may synergistically inhibit NSCLC growth and improve the efficacy of gefitinib. Given the biologic rationale for combining EGFR and VEGFR targeted agents and the encouraging results from previous studies, further exploration to evaluate the effectiveness of EGFR-TKIs in combination with antiangiogenic agents as first-line therapy in EGFR-mutation-positive NSCLC patients is merited.

In our former pilot phase I study, we have found that apatinib plus gefitinib had a tolerable safety profile and promising antitumor activity. The median PFS of the apatinib (500 mg) plus gefitinib (250 mg) group was 19.0 months, much longer than historically reported by gefitinib monotherapy [29]. This innovative design of apatinib plus gefitinib will enable patients to derive a survival benefit from the combinational regimen while convenience and comfort were taken into account.

As the first study aiming to evaluate EGFR-TKI plus VEGFR-TKI as a first-line therapy for advanced NSCLC, this ACTIVE phase III study will further assess the combination of apatinib and gefitinib as a new treatment approach for EGFR-mutant NSCLC in the first-line setting, with the aim to prolong survival and to delay the onset of resistance. The present trial will contribute to comprehensive information and evidence regarding the treatment of EGFR-TKIs combined with antiangiogenic therapy. If clinically meaningful efficacy results are observed and the primary endpoint is achieved, together with an acceptable safety and tolerability profile, findings from this study would demonstrate strong evidence of a new standard for first-line treatment of advanced NSCLC patients harboring activating EGFR mutations.

## Data Availability

The data supporting our trial will be found at the online Research Data Deposit website (http://www.researchdata.org.cn) after the trial database locked and fully analyzed.

## Abbreviations

EGFR-TKIs: epidermal growth factor receptor tyrosine kinase inhibitors
NSCLC: non-small cell lung cancer
VEGFR: vascular endothelial growth factor receptor
PFS: progression-free survival
OS: overall survival
ORR: objective response rate
DCR: disease control rate
TTPD: time to progressive disease
DoR: duration of response
QoL: quality of life
CFDA: China Food and Drug Administration
RECIST: Response Evaluation Criteria in Solid Tumors
CR: complete response
PR: partial response
SD: stable disease
PD: progressive disease
IRC: independent radiological central
SAEs: severe adverse events
AEs: adverse events
NCI-CTC: National Cancer Institute Common Toxicity Criteria
eCRF: electronic case report form
EDC: electronic data capture
